# Heterothermy as the Norm, Homeothermy as the Exception: Variable Torpor Patterns in the South American Marsupial Monito del Monte (*Dromiciops gliroides*)

**DOI:** 10.3389/fphys.2021.682394

**Published:** 2021-07-12

**Authors:** Roberto F. Nespolo, Carlos Mejías, Angelo Espinoza, Julián Quintero-Galvis, Enrico L. Rezende, Francisco E. Fontúrbel, Francisco Bozinovic

**Affiliations:** ^1^Instituto de Ciencias Ambientales y Evolutivas, Universidad Austral de Chile, Valdivia, Chile; ^2^Center of Applied Ecology and Sustainability (CAPES), Departamento de Ecología Facultad de Ciencias Biológicas, Pontificia Universidad Católica de Chile, Santiago, Chile; ^3^Millennium Institute for Integrative Biology (iBio), Santiago, Chile; ^4^Instituto de Biología, Pontificia Universidad Católica de Valparaíso, Valparaíso, Chile

**Keywords:** *Dromiciops gliroides*, hibernation, Microbiotheria, passive cooling, heterothermia, torpor, non-Holarctic heterotherms

## Abstract

Hibernation (i.e., multiday torpor) is considered an adaptive strategy of mammals to face seasonal environmental challenges such as food, cold, and/or water shortage. It has been considered functionally different from daily torpor, a physiological strategy to cope with unpredictable environments. However, recent studies have shown large variability in patterns of hibernation and daily torpor (“heterothermic responses”), especially in species from tropical and subtropical regions. The arboreal marsupial “monito del monte” (*Dromiciops gliroides*) is the last living representative of the order Microbiotheria and is known to express both short torpor episodes and also multiday torpor depending on environmental conditions. However, only limited laboratory experiments have documented these patterns in *D. gliroides*. Here, we combined laboratory and field experiments to characterize the heterothermic responses in this marsupial at extreme temperatures. We used intraperitoneal data loggers and simultaneous measurement of ambient and body temperatures (*T*_A_ and *T*_B_, respectively) for analyzing variations in the thermal differential, in active and torpid animals. We also explored how this differential was affected by environmental variables (*T*_A_, natural photoperiod changes, food availability, and body mass changes), using mixed-effects generalized linear models. Our results suggest that: (1) individuals express short bouts of torpor, independently of *T*_A_ and even during the reproductive period; (2) seasonal torpor also occurs in *D. gliroides*, with a maximum bout duration of 5 days and a mean defended *T*_B_ of 3.6 ± 0.9°C (one individual controlled *T*_B_ at 0.09°C, at sub-freezing *T*_A_); (3) the best model explaining torpor occurrence (Akaike information criteria weight = 0.59) discarded all predictor variables except for photoperiod and a photoperiod by food interaction. Altogether, these results confirm that this marsupial expresses a dynamic form of torpor that progresses from short torpor to hibernation as daylength shortens. These data add to a growing body of evidence characterizing tropical and sub-tropical heterothermy as a form of opportunistic torpor, expressed as daily or seasonal torpor depending on environmental conditions.

## Introduction

Many endotherms (i.e., birds and mammals) express transient periods of metabolic depression (known as “torpor” or “heterothermy”) as adaptations to seasonal or unpredictable changes in environmental productivity (Schmidt-Nielsen, 1979; Ruf and Geiser, 2015). When torpid, animal maintenance costs fall to a fraction of the resting metabolic rate (RMR), providing significant energy savings (up to 95%, according to Geiser, 2020). Thus, this should be the “logical” solution for animals that cannot migrate to better environments during seasons of resource scarcity (Schmidt-Nielsen, 1979). The metabolic depression during torpor can be profound, with body temperatures (*T*_B_) approaching ambient temperature (*T*_A_) and torpor bout duration of several days or weeks. This has been classically referred as “hibernation” or “seasonal torpor” (Geiser and Heldmaier, 1995; Heldmaier et al., 2004; Geiser, 2020; Nowack et al., 2020). Nevertheless, several species also express short episodes of heterothermy, lasting a few hours or a day, called “daily torpor”.

In the southern hemisphere, it was estimated that 43% of the Australian mammal species exhibit some form of heterothermy (Geiser and Körtner, 2010), showing a large physiological diversity, ranging from the long hibernation of echidnas (7 months) to the short torpor bouts of Dasyurid marsupials and bats (Geiser and Körtner, 2010). In these animals, the metabolic reduction of torpor ranges from 29.5% of basal metabolic rate in daily heterotherms (*T*_B_ reductions of ~20°C) to 5.1% in hibernators (*T*_B_ reductions of ~30°C, see Geiser and Ruf, 1995). Interestingly, in hibernators even short torpor bouts elicit these important metabolic reductions. On the other hand, African heterotherms include Madagascar tenrecs (*Echinops telfari*), displaying seasonal torpor (Lovegrove and Genin, 2008), and elephant shrews (*Elephantulus* spp.) expressing characteristics of daily torpor (bout duration) and hibernation (metabolic reduction; Lovegrove et al., 2001). Cheirogaleidae lemurs (Ortmann et al., 1997) express daily torpor even at “warm” temperatures, and the mouse lemur (*Microcebus murinus*) showing flexible heterothermic phenotypes (Schmid and Ganzhorn, 2009).

Although torpor has been described on a few South American species of birds and mammals (hummingbirds: Carpenter, 1974; Wolf et al., 2020; bats: Bozinovic et al., 1985; rodents: Bozinovic and Marquet, 1991; didelphid marsupials: Bozinovic et al., 2007), multiday torpor was only described in the Patagonian opossum (*Lestodelphys halli*; which can attain a torpor duration of up to 42.5 h, see Geiser and Martin, 2013), and in the Microbiotheriid “monito del monte” (*Dromiciops gliroides*) known to express torpor bouts of up to 120 h (Bozinovic et al., 2004). However, the absence of systematic studies characterizing naturally occurring torpor in South American endotherms is notable, especially from the Neotropical region.

Here, we analyzed the natural heterothermic patterns of *D. gliroides* (“monitos,” hereafter), which have remained unclear since the first description by Bozinovic et al. (2004), mainly because all evidence on this species come from short-term laboratory trials (Bozinovic et al., 2004; Nespolo et al., 2010; Franco et al., 2017). For example, in a recent review, monitos are described as hibernating “apparently mainly in winter” (Geiser, 2020, p. 9). However, the same year other authors classified monitos as opportunistic heterotherms that enter torpor under long photoperiod and relatively warm temperatures, even when food is available (Nowack et al., 2020, p. 17). Thus, to clarify this information and explore how this marsupial performs at extreme temperatures, we performed this study, combining laboratory and field experiments. We believe that this is important as, besides being one of the few South American mammals described as hibernator, monitos are a paradigmatic species (see below).

Today, living South American marsupials represent nearly 10% of the mammalian fauna of South America and comprise about 100 species grouped in the orders Didelphimorphia, Paucituberculata, and the relict order Microbiotheria, which is represented by the sole living species *D. gliroides* (Goin et al., 2016). Microbiotheria was a marsupial order that comprised at least 14 species that dominated southern South America in the Eocene–Miocene, encompassing a wide geographic range that extended from present day Bolivia to Antarctica (Hershkovitz, 1999; Goin and Abello, 2013; Goin et al., 2016). Abundant fossil evidence, as well as current *Dromiciops* biology, suggests that Microbiotheriids were intimately associated with the neotropical temperate forests, forming the so-called *Microbiothere–Chusquea–Nothofagus* association (Hershkovitz, 1999). Also, Microbiotheria are known to be the sister group of Australidelphia (Australian marsupials), which colonized Australia through an Antarctic bridge during the Cretaceous (Nilsson et al., 2010; Mitchell et al., 2014). Then, *Dromiciops* could be considered as the Australidelphian ancestral model, which evolved and diversified into forms as diverse as koalas, wallabies, and Tasmanian devils (Nilsson et al., 2010; Mitchell et al., 2014; Goin et al., 2016).

Monitos are presently distributed over a broad geographic range (ca. 900 km north to south), including Andean forests where freezing temperatures are common in winter (e.g., Altos de Lircay and Futaleufú, see Oda et al., 2019; Mejias et al., 2021). In these locations, monitos probably hibernate at temperatures close to zero. But also, monitos are present in populations where summer temperatures surpass 30°C, in the northern edge of the range (=35°S, see Uribe et al., 2017). In the laboratory, monitos trigger their heterothermic state primarily when fasted, and torpor episodes are deeper and longer under cold conditions (Nespolo et al., 2010, 2021). The transition from homeothermy to heterothermy in the monitos is characterized by passive cooling after a sudden reduction in metabolic rate, which takes about 5–6 h depending on *T*_A_ (Cortes et al., 2014). It is unknown if monitos defend a minimum *T*_B_ and thermoregulate in torpor and if they arouse at some critical *T*_A_.

**Figure 1:**
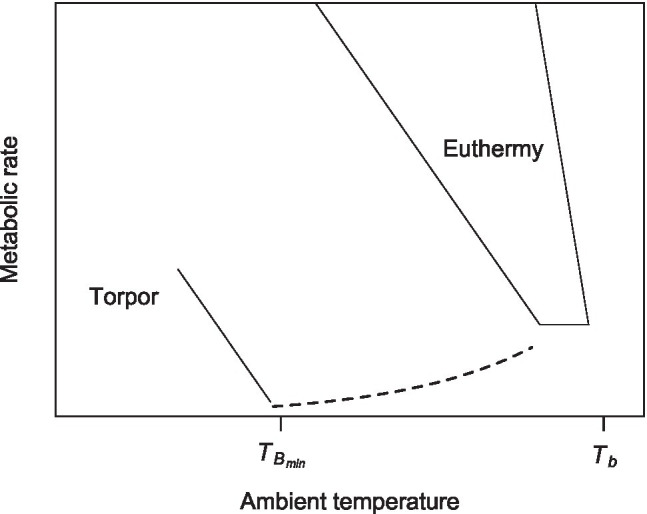
A thermoregulatory polygon of a hibernator, showing the metabolic curve of torpor and the critical body temperature where animals start thermoregulating in torpor (*T*_B_min; see text for details, modified from Rezende and Bacigalupe, 2015).

The control of *T*_B_ during heterothermia (“torpor thermoregulation,” hereafter) was first described by (Hainsworth and Wolf, 1970), in the hummingbird *Eulampis jugularis*, and is represented in the metabolic curve by a negative slope line running below (and parallel) to the euthermic metabolic curve (Figure 1; Rezende and Bacigalupe, 2015).

Then, below a certain critical *T*_A_ (=*T*_Bmin_), the torpid animal stops passive cooling (represented by the dotted line in Figure 1) and begins metabolic heat production, maintaining constant *T*_B_. Torpor thermoregulation has been subsequently described in several species of mammals (Geiser and Baudinette, 1987; Richter et al., 2015) and birds (Hiebert, 1990; Wolf et al., 2020).

In this study we analyzed a suite of torpor thermoregulatory patterns in *D. gliroides*, by recording *T*_A_ and *T*_B_ using miniature data loggers in individuals under field and laboratory conditions. In addition, we used the *T*_B_ − *T*_A_ thermal differential to estimate the environmental factors that best explain the field occurrence of torpor. We hypothesized that: (1) *D. gliroides* is an opportunistic hibernator that could express short and multiday torpor depending on environmental conditions; (2) short torpor shifts into multiday torpor as the cold season progresses, primarily induced by shortening daylength and reduced food availability, and (3) *D. gliroides* has a critical torpor temperature slightly higher than zero degrees, thereby thermoregulating to control its T_B_ above freezing temperature.

## Materials and Methods

### Animals

All procedures presented in this study were approved by the Chilean Agriculture and Livestock Bureau (SAG; permits No 4371/2019 and 3393/2019) and by the Bioethics Committee of the Austral University of Chile (resolution 313/2018 annex 2019). We performed the experiments in eight *D. gliroides* individuals (*M*_B_ range: 26.1–54.8 g, five females, three males) over 2 years. Animals were captured in San Martin Biological Station (39°38'50.71' S 73°11'46.43' O) in February 2019. No animal was harmed in these procedures, the data loggers were safely removed, and all animals were released at the place of capture after finishing the study. We followed the guidelines of the American Society of Mammalogists for the use of wild mammals in research (Gannon et al., 2007; Sikes et al., 2011). Since we only had four functional data loggers, experiments had to be done in different batches for reusing the devices.

**Figure 2:**
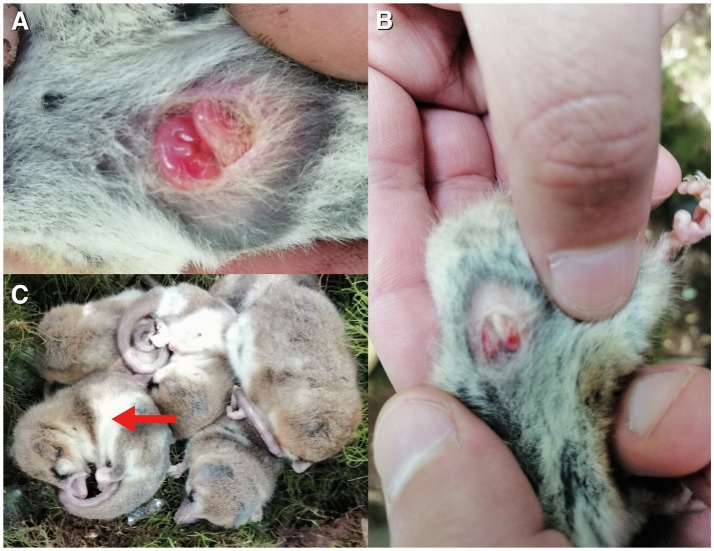
**(A)** torpid pups (*n* = 2, approximate age: 3 weeks) found in a torpid female (*M*_B_ = 25 g) on December 18, 2020, in the field, with *T*_A_ = 25°C and *T*_B_ of the female = 25.1°C. Pups were not moving. **(B)** shows another view, and **(C)** shows the cluster of torpid individuals found this day (red arrow denotes the examined female). All these animals were found active the next day.

We included data from 2019 where several individuals (*n* = 25) were captured in San Martin in February and maintained in enclosures, for another study. In one of the enclosures this year (with three males and two females), one of the females gave birth to two pups of approximately 4 mm, which were discovered on December 10 (approximate age: 2 weeks; Muñoz-Pedreros et al., 2005). 1 week later (December 17, 12 am, *T*_A_ = 25°C), we found the whole cluster of five animals torpid, including the female and the two pups (cloacal *T*_B_ = 25.3 ± 0.4°C, mean ± SE; Figure 2).

We included these results in this paper, as it contributes to the idea that *D. gliroides* routinely perform torpor independently of the season and even during the reproductive period. We later released the female with the pups at the site of capture.

### Datalogger Deployment

We surgically implanted body temperature (*T*_B_) dataloggers (model DST nano, 1.3 g, cylindric, 17 mm long, 6 mm diameter; Star Oddi, Iceland) into the abdomen (i.e., intraperitoneal) of four individuals in March 2019 (autumn) and removed them in November (spring). According to the manufacturer, the devices are calibrated at factory over a temperature range of −10–50°C. We calibrated the devices in a beaker with water at 40°C that was allowed to cool to room temperature (10°C), with temperature records every 2 min, using a laboratory thermometer (alcohol). The linear regression between water and logger temperature (20 points) was highly significant (*R*^2^ = 0.99, *p* = 0.001). We set the dataloggers for hourly recordings during the first winter. The next winter, we reused them in other two animals for one-month ramp experiments designed to determine torpor patterns at a broad thermal range (see details in the “ramp experiment” section below). We removed dataloggers from these animals after 1 month of continuous measurements. For both implantation and removal, we used subcutaneous tramadol 5 mg kg^−1^, plus inhalation anesthesia for induction (isoflurane in oxygen 5%) and maintenance (isoflurane in oxygen 2.5%). We then administered subcutaneous meloxicam 0.5 mg kg^−1^. We have previously sterilized the dataloggers with formaldehyde gas and UV exposure for 15 min and then set them to record temperature hourly. The surgical approach consisted in a small incision (3 mm) on the abdominal region in their median plane. The device was delicately placed perpendicularly to the body axis, between the layers of the peritoneum. The wound was closed with a stitch using sutures that are self-absorbing, both in the muscular plane and in the skin. Every time, we maintained the animals in the clinic for 2 weeks for recovery after the surgery. No animal was harmed in any of these surgeries. Surgical procedures had a duration of 5 min per animal and were led by the third author (AE), an expert veterinarian in charge of a center for wildlife recovery (CEREFAS-UACh).

### Enclosures and Outdoor Experiment

**Figure 3:**
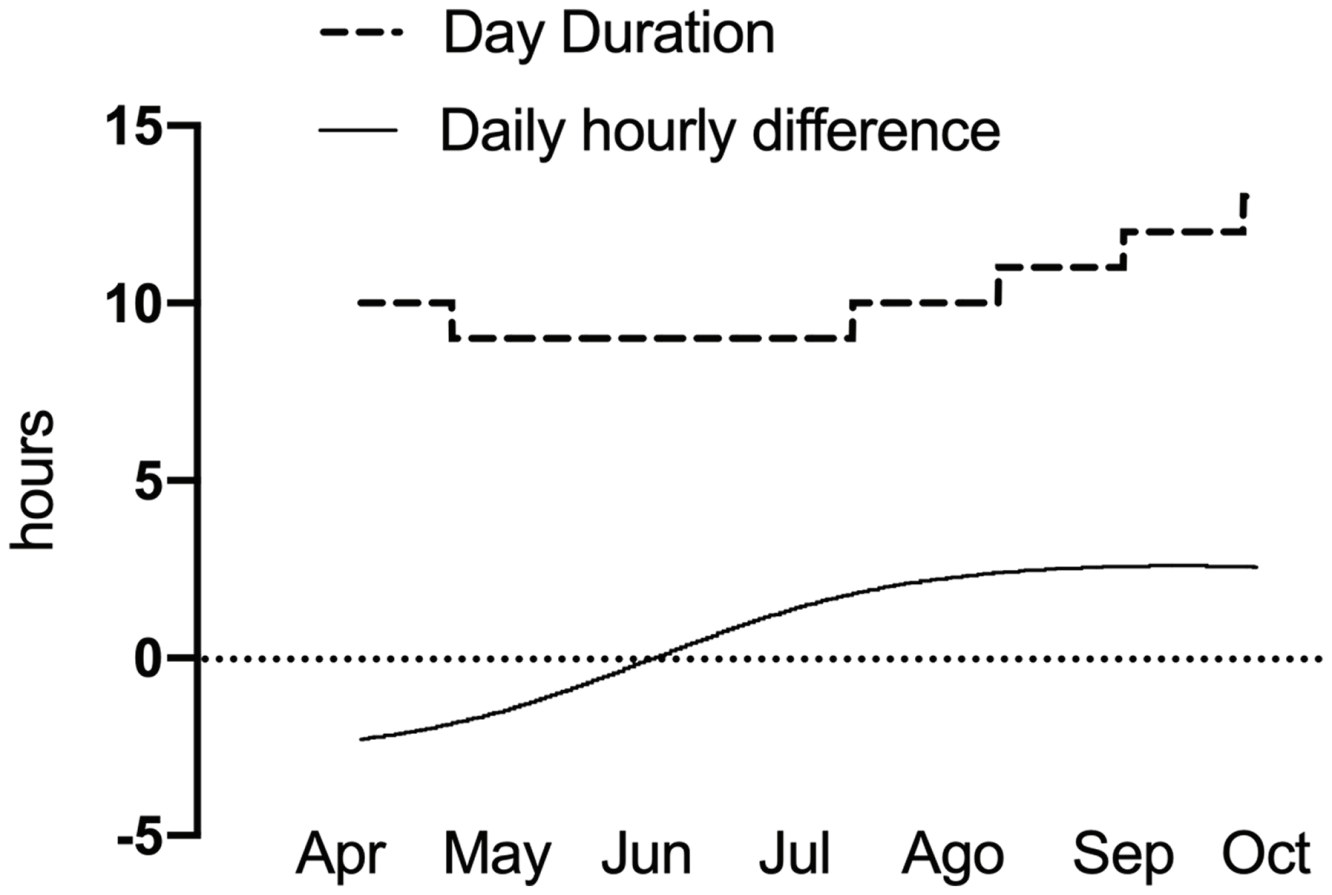
Natural photoperiodic changes during the study period in the outdoor enclosures.

We installed four enclosures in San Martin Biological Station, specifically in an area of dense forest and abundant understory, typical of *D. gliroides* habitat (Fonturbel et al., 2012). Each enclosure is cylindrical (~2m^3^ of volume) and included twigs and plant material mimicking this species’ microenvironment. It had a mesh that allows light and ventilation to enter a similar way under the forest canopy, checked with a luxmeter (TASI 21^®^). We also included one temperature datalogger (HOBO^®^, Onset, United States) set for recording air temperature (*T*_A_) every 15 min and located at 1 m above ground in the shadow. During the outdoor experiment (27 weeks, from April to October 2019), we recorded a mean monsthly *T*_A_ ranged from 17.1°C_max_ and 3°C_min_ (the T_A_ range for one of the enclosures is shown in Results), and the seasonal change in photoperiod was 4.5 h (Figure 3).

Water was provided *ad libitum* in a plastic plate (1 L). Each individual was released in one different enclosure, where food was provided daily: two of the animals received “restricted” food (20 kJ day^−1^ per animal), and the other two received *ad libitum* food (100 kJ day^−1^ per animal). Food consisted of a mix of cereals, jam, and tuna that provides balanced nutrition for this omnivorous species (Contreras et al., 2014). Energy content was determined using a calorimeter (Parr Instruments, IL, United States). This experiment is further referred to as the “*outdoor experiment,*” which aimed to determine how photoperiod, *T*_A_, food availability, and body condition induced metabolic reductions. During this experiment, we gently weighed the animals on a weekly basis to determine their body mass (*M*_B_) and body mass change from the last week. We replaced food plates every day to ensure animals received the constant amounts.

### Ramp Experiment

Given that some of the dataloggers still had operational batteries, we reused them in the 2020 winter, with two newly captured animals in a controlled *“ramp experiment”* to determine torpor at near-zero *T*_A_. We performed the ramp experiment (see similar experiments in Currie et al., 2015), on August 1, 2020, in a Pelt-5 incubator and temperature controller (Sable Systems) set at the mean *T*_A_ of the moment (winter, mean *T*_A_ = 5.0 ± 2.1°C) and photoperiod accordingly (10,14, see Figure 3). We programmed the incubator for a gradual reduction of 1°C per day, passing one day at a stable *T*_A_ until −2.4°C (this limit given by the equipment). In a few cases, the cooling system froze, raising the set point’s temperature, which did not produce a perfect ramp. Animals did not arouse during these short *T*_A_ increments. During the ramp experiment, we offered water *ad libitum* but removed food. Also, we maintained animals in separated cages within the incubator. After the “cold” ramp, animals remained in the incubator at 15°C for 1 week. Then, we assayed the “warm ramp,” which consisted of a similar ramping rate as before but increasing temperature from 15°C until 28°C. No animals were harmed during these ramps and were released in the capture site in spring (September 21, 2020) after checking their physiological condition by a trained veterinarian.

### Statistical Analyses

To calculate steady-state torpor temperature (i.e., the critical body temperature where animals start thermoregulating in torpor, *T*_Bmin_), we performed linear regressions between the thermal differential (i.e., *T*_B_ − *T*_A_; *T*_DIFF_) and *T*_A_ with the data points of the plot where *T*_DIFF_ is inversely proportional to *T*_A_ during torpor. That is, we amplified each graph and took every point that fell in the bottom-left edge of the figure. To estimate torpor thermoregulation regressions, we predicted the RMR as RMR = *C*_min_(*T*_B_ − *T*_A_; =Newton’s model for passive cooling, see Rezende and Bacigalupe, 2015); where Cmin is minimum thermal conductance (=3.4848 J g^−1^°C^−1^ h^−1^, see Bozinovic et al., 2004).

The four individuals used for the outdoor experiment produced hourly records of *T*_DIFF_ for 25 weeks (=10,533 observations, determined by the data loggers’ battery capacity). Then, to determine the combination of variables that trigger torpor, we used *T*_DIFF_ as a continuous response variable associated with fixed and random factors. We used *T*_DIFF_ because we got non-convergent models when using it as a dichotomic variable (i.e., active or torpor). Each record was associated with the fixed factors: food (low/high), *T*_A_ (°C), photoperiod (daylength in hours), delta photoperiod (difference in daylength from the day before in hours), *M*_B_ (g), and ∆*M*_B_ (difference *M*_B_ with the previous week). Then, we fitted mixed-effects generalized linear models (GLMM) with a Gaussian error distribution, including individual ID, sampling week, and date as random effects to account for inter-individual and inter-enclosure variability along with the repeated measures in time (Zuur et al., 2009). We used the Akaike information criteria (AIC) to select the best models and contrast the candidate models’ performance. We dropped *M*_B_ and delta photoperiod for being redundant with photoperiod and ∆*M*_B_. All combinations of models were tested first, including all possible interactions (not presented). Here, we present the best nine models:

→ Model 1: *T*_DIFF_ ~ food + (1 | ID) + (1 | Date)
→ Model 2: *T*_DIFF_ ~ food + *T*_A_ + (1 | week) + (1 | ID) + (1 | Date)
→ Model 3: *T*_DIFF_ ~ food + *T*_A_ + photoperiod + (1 | week) + (1 | ID) + (1 | Date)
→ Model 4: *T*_DIFF_ ~ food + *T*_A_ + photoperiod + ∆M_B_ + (1 | week) + (1 | ID) + (1 | Date)
→ Model 5: *T*_DIFF_ ~ food × *T*_A_ + (1 | week) + (1 | ID) + (1 | Date)
→ Model 6: *T*_DIFF_ ~ food × photoperiod + (1 | week) + (1 | ID) + (1 | Date)
→ Model 7: *T*_DIFF_ ~ *T*_A_ × photoperiod + (1 | week) + (1 | ID) + (1 | Date)
→ Model 8: *T*_DIFF_ ~ *T*_A_ × ∆*M*_B_ + (1 | week) + (1 | ID) + (1 | Date)
→ Model 9: *T*_DIFF_ ~ photoperiod × ∆*M*_B_ + (1 | week) + (1 | ID) + (1 | Date)

The model including all the interactions was discarded since it gave a variance inflation factor (VIF) = 2048.

We estimated GLMM parameters and their significance using a restricted maximum likelihood approach with a Kenward–Roger approximation to estimate degrees of freedom (Halekoh and Hojsgaard, 2014). We performed all analyses using R version 3.6.0 (Crawley, 2007), with the packages mgcv (Wood, 2011), lme4 (Bates et al., 2013), lmerTest (Kusnetzova et al., 2015), and pbkrtest (Halekoh and Hojsgaard, 2014). We estimated the VIF value from the fitted GLMM models to control for multicollinearity problems. VIF values >3 represent a high multicollinearity problem. From the models listed above, only models 7, 8, and 9 resulted in VIF values above 3.

## Results

We detected a mean of 150 torpor bouts of 13.4 h of duration in four individuals at the outdoor experiment, with interbout euthermic periods of 13.6 h (Table 1).

**Table 1:** Hibernation parameters (mean ± SE) of monito del monte (*Dromiciops gliroides*) obtained in this study under natural conditions (*n* = 4; this excludes the ramp experiments).

Hibernation paramater	Mean ± SE	max	min
Hibernation duration (h)	4,053 ± 30.7	4,132	3,999
Number of torpor bouts	150 ± 5.1	162	139
Mean duration of torpor bouts (h)	13.4 ± 0.83	124	1
Mean duration of IBE (h)	13.6 ± 1.2	116	2
Minimal body temperature (*T*_B_°C)	4.13 ± 0.24	4.8	3.7

Monitos express torpor routinely, sometimes on summer days at *T*_A_ = 20°C (Figure 5A) and in summer at cool *T*_A_ (15°C, Figure 5B).

**Figure 4:**
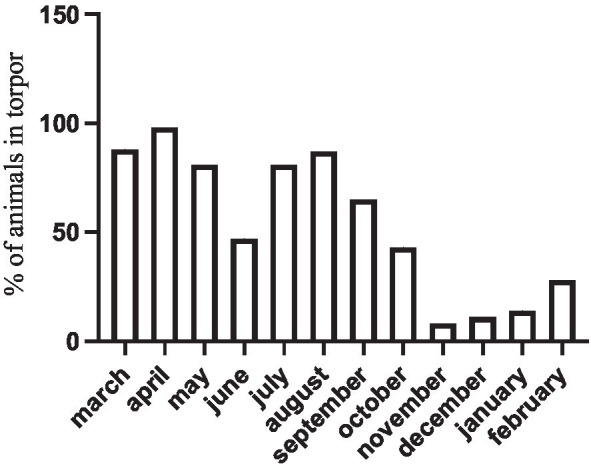
Percentage of torpid *D. gliroides* (*n* = 25) in a field study using outdoor enclosures. Torpor incidence was recorded weekly.

These representative cases are also supported by extensive observations of yearly torpor occurrence in a companion field study (Nespolo et al., 2021). In this study, we recorded a monthly torpor incidence (i.e., the number torpid individuals found in weekly visits, over the total sample of *n* = 25 individuals) that increased in winter, but was never zero even in the warmest days of the summer (Figure 4).

**Figure 5:**
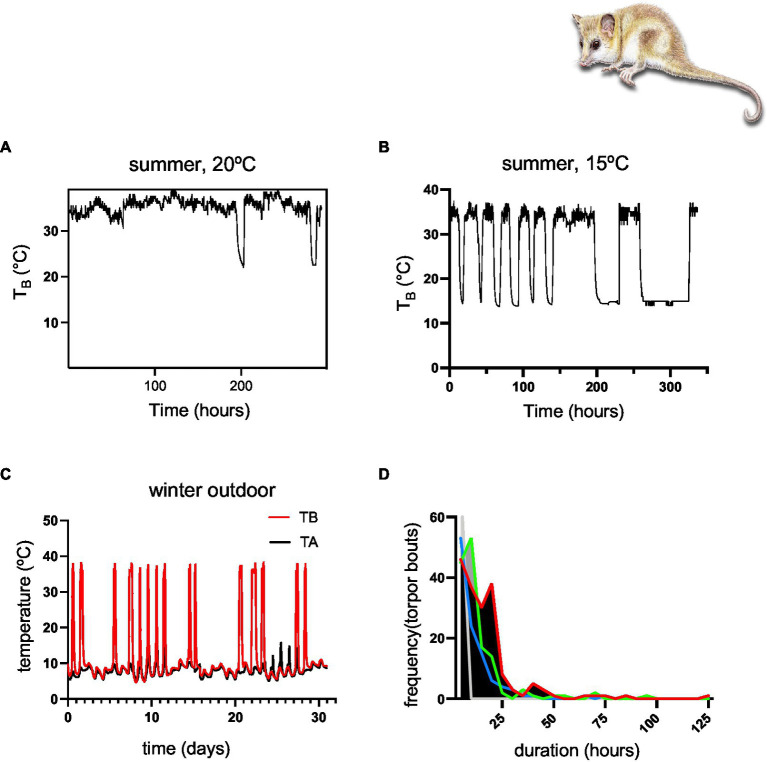
**(A)** Summer experiments in climatic chambers showing short torpor episodes at *T*_A_ = 20°C, **(B)** summer experiment in climatic chambers showing torpor episodes at *T*_A_ = 15°C, **(C)** a representative record of one heterothermic individual during winter (August), showing a few multiday torpor episodes of several days and frequent periodic arousals, **(D)** statistics of the outdoor experiment showing a high frequency of short torpor episodes of 10 h, and some long, multiday torpor episodes of 100–125 h (detailed statistics are shown in Table 1; colors represent different individuals).

Moreover, monitos enter torpor even during the reproductive period, including females with pups (see Figure 2). However, winter torpor is more frequent, and torpor bouts are longer (Figure 5C), lasting up to 125 h in some cases (*cf.* Figure 5D; Table 1).

**Figure 6:**
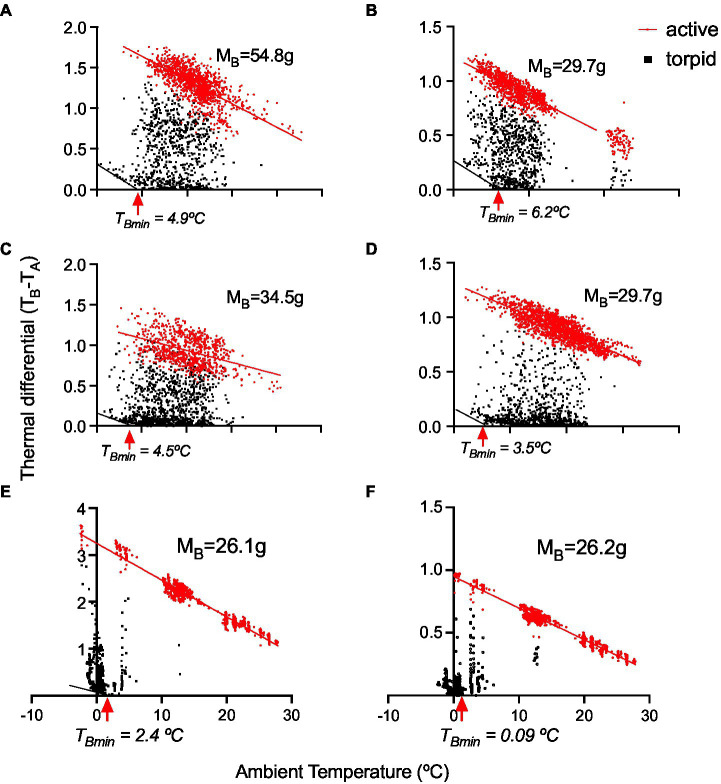
Thermal differential (*T*_B_ − *T*_A_) in six monitos. The first four animals **(A–D)** are from the outdoor experiment, and the last two **(E,F)** were from the ramp experiment, showing torpor at sub-freezing *T*_A_s (see the details in Figure 8). *T*_Bmin_ denotes the temperature at which animals start thermoregulating.

The variable torpor patterns are also reflected in T_DIFF_ (Figure 6), from which we calculated the critical torpor temperature (*T*_Bmin_) below, in which torpid animals start thermoregulating (mean *T*_Bmin_ = 3.6 ± 0.9°C, *n* = 6; Figure 6).

**Figure 7:**
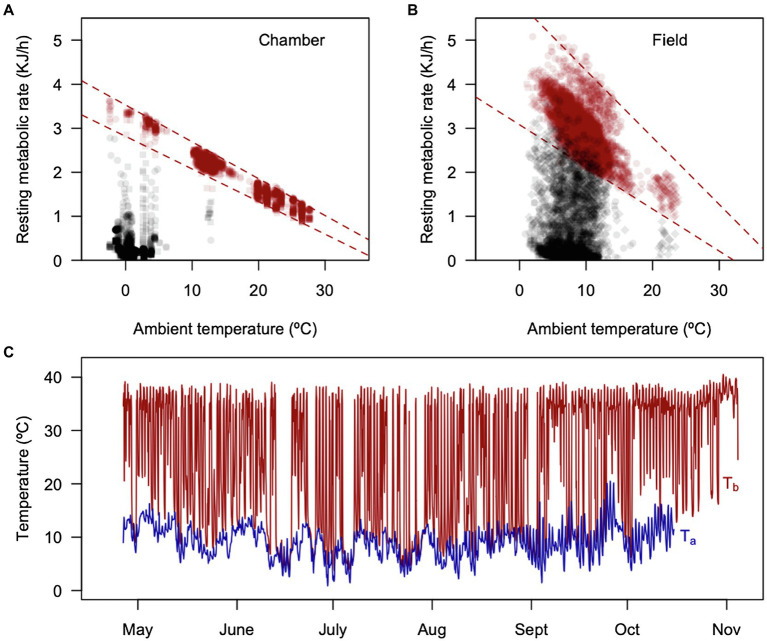
Summaries of calculated RMRs from Figure 5, using Newton’s equation for passive cooling: RMR = *C*_min_(*T*_B_ − *T*_A_), where *C*_min_ = 3.4848 Jg^−1^ h^−1^°C^−1^ (Bozinovic et al., 2004), comparing the metabolic variability obtained in a climatic chamber **(A)** ramp experiments and in the field **(B)** outdoor experiments. **(C)** Shows the ambient and body temperature of a representative individual during the outdoor experiment.

The torpor thermoregulation regression for the pooled sample (six individuals with data loggers) was RMR(kJh^−1^) = 0.62 ± 0.2 (kJh^−1^) – 0.15 ± 0.03 × *T*_A_ (kJh^−1^°C^−1^; all regressions were significant, with *R*^2^ values ranging from 0.76 to 0.97). This implies that torpid individuals, once reaching the critical *T*_B_ of 3.6°C, start generating 0.15 kJ per hour per every 1°C reduction. These RMR patterns were quite less variable in the chamber experiment (summarized in Figure 7A), compared with the field (Figure 7B). A representative whole-season pattern of torpor is presented in Figure 7C, as a reference.

**Figure 8:**
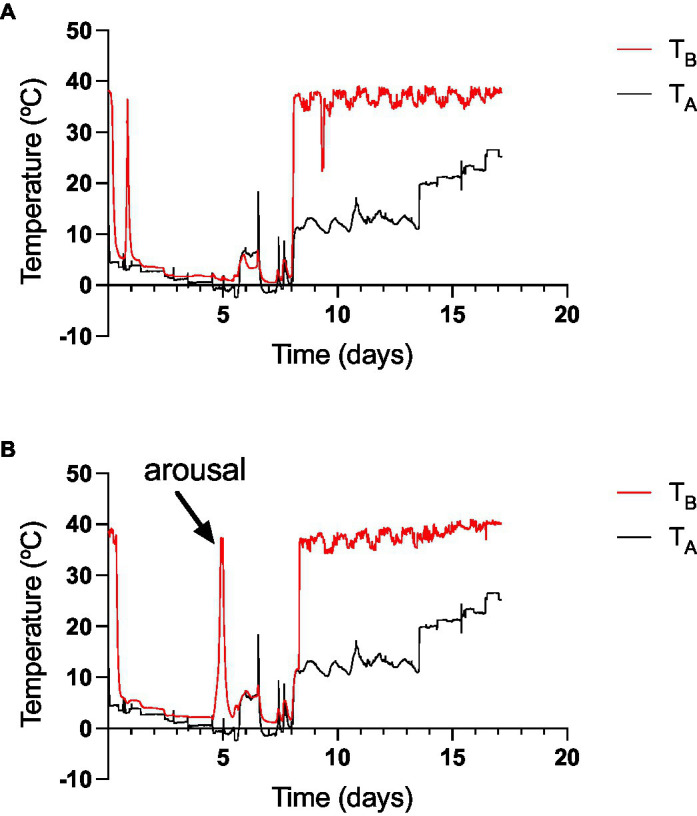
The ramping experiment, where two *D. gliroides* (**A,B**; without food access), with intraperitoneal data loggers were gradually exposed first to diminishing temperatures from 5°C to −2.4°C (rate: 1°C day^−1^). At day 8, *T*_A_ was elevated gradually at a similar rate until 28°C, and food was provided *ad libitum*. The spikes in *T*_A_ are because chamber openings. The elevation of *T*_A_ between the sixth and seventh day occurred because of the freezing of the climatic chamber compressor.

The ramp experiments’ details, where two monitos were taken to the extreme *T*_A_ of −2.4 degrees during two periods of 24 h and then gradually returned to room temperature with food and water *ad libitum*, are presented in Figure 8. In these trials, animals entered torpor within the first 12 h of the experiment, and one individual aroused from torpor when *T*_A_s felt below zero, it remained euthermic for 24 h, and then it entered torpor again (Figure 8B). The other animal remained in torpor during the whole two periods at sub-zero *T*_A_ (Figure 8A). Both individuals maintained their *T*_B_ slightly above zero when experiencing sub-freezing temperatures. As indicated before, the first animal had a *T*_Bmin_ of 2.4 and the other had a *T*_Bmin_ 0.09°C (see Figures 6E,F). Both animals aroused from torpor when *T*_A_ was increased above 10°C, and food was put back in the chamber.

The multi-model analysis indicated that the most important variable explaining torpor occurrence (measured as reductions in *T*_DIFF_) is a combination of food and photoperiod (Table 2: *ω*AIC = 0.59).

**Table 2:** Multi-model selection results upon nine candidate models based on Akaike information criteria (AICc). Multicollinearity was tested using the maximum value of the variance inflation factor (VIF) for each model.

Model	Random	df	AICc	∆AICc	*ω*AIC	max VIF
*T*_DIFF_ ~ food × photoperiod	w + I + D	8	35666.1	0.00	0.590	1.644
*T*_DIFF_ ~ photoperiod × ∆*M*_B_	w + I + D	8	35667.7	1.59	0.266	70.905
*T*_DIFF_ ~ food × *T*_A_	w + I + D	8	35669.0	2.94	0.136	1.435
*T*_DIFF_ ~ food + *T*_A_ + photoperiod + ∆*M*_B_	w + I + D	9	35674.6	8.55	0.008	1.043
*T*_DIFF_ ~ *T*_A_ × ∆*M*_B_	w + I + D	8	35700.1	34.05	<0.001	5.717
*T*_DIFF_ ~ food + *T*_A_ + photoperiod	w + I + D	8	35702.9	36.85	<0.001	1.039
*T*_DIFF_ ~ *T*_A_ × photoperiod	w + I + D	8	35708.2	42.15	<0.001	76.463
*T*_DIFF_ ~ food + *T*_A_	w + I + D	7	35723.4	57.32	<0.001	1.001
*T*_DIFF_ ~ food	I + D	5	36759.5	1093.40	<0.001	1.000

RMR, resting metabolic rate; food = calorie restriction treatment (restricted or *ad libitum*), photoperiod = photoperiod (number of light hours per day), ∆*M*_B_ = weekly change in body mass, *T*_A_ = ambient temperature. Random factors were week (w), ID (I), and date (D).

Body mass reductions (∆*M*_B_) and photoperiodic changes were dropped from the models, thus not significantly contributing to the observed variation in *T*_DIFF_. According to the obtained coefficients, the most important factor explaining torpor occurrence was photoperiod’s main effect, with a coefficient of 9.9 ± 1.5 (*p* < 0.001, Table 3). The positive value of this coefficient suggests that longer days are associated with higher T_DIFF_. This term was followed by the interaction between photoperiod and restricted food (coefficient: −3.2 ± 1.5, *p* < 0.01, Table 3), whose negative sign suggests that the response to photoperiod was stronger when food was restricted than when provided *ad libitum*. Food supply alone was not a significant contributor to the observed variation in *T*_DIFF_ (*p* = 0.55; Table 3).

**Table 3:** Estimates of the fixed effects from the mixed-effects generalized linear models (the best model chosen after multi-model selection, see Table 2) used to assess the determinant factors on thermal differential (*T*_DIFF_).

Fixed effects	Coefficient ± SE	*t*	*P*
Intercept	−1.352 ± 0.767	−1.762	0.090
Food (restricted)	0.410 ± 0.603	0.679	0.545
Photoperiod	9.935 ± 1.493	6.653	<0.001
Food (res) × Photoperiod	−3.237 ± 0.608	−5.320	<0.001

Reference levels for categorical predictors are in parenthesis. Random effects were: week, date, and individual ID (non-significant).

## Discussion

The comparison of monitos heterothermic patterns with other species suggest a dynamic form of heterothermy that in some respects is similar to daily torpor (i.e., short bouts of less than 24 h, summarized in Ruf and Geiser, 2015). Also, monitos do not restrict their torpor episodes to the cold season (Figure 3); and show a high frequency of arousals during winter (Figure 3C; Table 1). On the other hand, monitos exhibited a torpid *T*_B_ that was almost indistinguishable from *T*_A_ (Figure 3), with an extreme of 4.13°C in the outdoor experiment and 0.08°C in the climatic chamber (Figure 5), where animals endured sub-freezing temperatures (Figure 8). These minimum torpid *T*_B_ are considerably lower than what is observed in hibernators such as the garden dormouse (=6°C, from Table 1 in Giroud et al., 2021) and lower than what is reported for most Holarctic and non-Holarctic heterotherms (see Figure 3 in Nowack et al., 2020). Similarly, torpor bout duration reached a maximum of 5.2 days (125 h, Figure 3), which is coincident with the first report of Bozinovic et al. (2004) in the laboratory (=5.0 days). Thus, applying the criteria of Geiser (2020): “hibernators are defined as species that can express multiday torpor of >2 days, whereas daily heterotherms are defined as species expressing daily torpor exclusively under all thermal, environmental and nutritional conditions”; monitos would fall into the seasonal torpor category.

We observed torpid monitos at *T*_A_ = 25°C, including females with pups, which suggests these animals express torpor routinely, irrespective of *T*_A_ (see also Figure 5B). This is coincident with what was observed in heterothermic birds and mammals in unpredictable environments (e.g., hummingbirds and nig=htjars), which express torpor when incubating and also in mammals of the three orders (i.e., monotremes, marsupials, and placentals), which express torpor during pregnancy and lactation (reviewed in McAllan and Geiser, 2014). Similarly, torpor at room temperatures is known to occur in rodents (Grimpo et al., 2014), bats (Johnson and Lacki, 2014), and primates (Hadj-Moussa and Storey, 2019) as adaptations to seasonally dry habitats. What is intriguing with *D. gliroides* is that it lives in a temperate rainforest characterized by humid and mild temperatures, where the water economy does not seem to be a problem. In fact, evaporative water loss measurements in torpid *D. gliroides* suggest water economy is comparatively poor in this species (Withers et al., 2012). Then, monitos seem to express heterothermia routinely, and regardless of *T*_A_.

But also, monitos exhibited endurance to cold. In fact, according to our ramp experiments, monitos reached a *T*_Bmin_ of 3.6°C (one individual had a *T*_Bmin_ = 0.08°C) and endured sub-freezing temperatures during 24 h. These individuals generated 0.15 kJh^−1^, per every Celsius degree reduced (i.e., the negative slope of the torpor thermoregulation regression). In daily heterotherms for instance, these parameters are *T*_Bmin_ = 8.3°C, and the slope of thermoregulation regression = 0.004 kJh^−1^°C^−1^ for the Rufous-tailed hummingbird (*Selasphorus rufus*, *M*_B_ ~ 3.3 g; Hiebert, 1990). On the other hand, for fat-tailed dunnarts (*Sminthopsis crassicaudata*) in winter, *T*_Bmin_ = 11°C and the thermoregulatory slope is 0.10 kJh^−1^°C^−1^ (Geiser and Baudinette, 1987). In the Arctic ground squirrel (*Urocitellus parryii*, *M*_B_ ~ 600 g), which is considered an extreme hibernator, the slope of the thermoregulatory regression was 0.17 kJh^−1^°C^−1^ (transformed to joules from Figure 1 at Richter et al., 2015), with *T*_Bmin_ = −3°C (Richter et al., 2015). Then, a thermoregulatory slope of 0.15 kJh^−1^°C^−1^ and *T*_Bmin_ = 3.6°C seems to be outstanding for a marsupial of temperate regions. Importantly, monitos are found in a relatively broad geographic area, including high Andean locations such as Altos de Lircay (the Northern edge of the distribution of *D. gliroides*), Futaleufú in Chile, and Parque Nacional Los Alerces in Argentina (latitude 43° South; Gurovich et al., 2015; Oda et al., 2019), where hibernacula are probably covered by snow during the winter (Honorato et al., 2016). Then, these capabilities are probably important in these high-altitude locations.

Previously, it was suggested that food deprivation combined with cold is important for inducing torpor in monitos (Nespolo et al., 2010), but it was unknown how these daily heterotherm patterns shifts into multiday torpor patterns in winter. Our results suggest that this shift is mainly driven by gradual reductions in the photoperiod, enhanced by reduced food availability (which, in our results, interacted with photoperiod). In general, it is known that experimental food reductions can modify seasonal torpor patterns in hibernators; either generating a shorter hibernation period or inducing shallower torpor episodes (reviewed in Vuarin et al., 2015). This dependence is mechanistically associated with a hypothalamic neuronal circuit stimulated by fasting that lowers the set point for *T*_B_ control, a phenomenon known for decades (Heller and Colliver, 1974; Geiser et al., 1990), and recently confirmed at the molecular level in laboratory mice (Hrvatin et al., 2020; Takahashi et al., 2020). In the case of *D. gliroides*, animals can sense the presence of food in the environment and adjust their energy expenditure (by torpor) on a weekly basis (Giroud et al., 2021; Nespolo et al., 2021). This modulation also occurs in other mammalian species of the southern hemisphere, such as African elephant shrews, which show daily heterothermy modulated by food, *T*_A_, and photoperiod (Mzilikazi and Lovegrove, 2004); in the short-beaked echidnas, which can shift from short torpor to hibernation depending on habitat conditions (Nicol and Andersen, 2007); in the marsupial *Antechinus*, which intensify torpor use under environmental threats such as food scarcity and fires (Stawski et al., 2016); and in spiny mice, which react with heterothermy in response to storms of floods (reviewed in Nowack et al., 2020). Thus, the opportunistic heterothermic patterns of this Neotropical marsupial then seem to concur with other non-Holarctic species’ heterothermic patterns.

Simple bioenergetic models have been successfully applied for calculating hibernation energy requirements using fat storages in the bat *Myotis lucifugus* (Humphries et al., 2002), for testing contrasting torpor hypotheses in the bat *Myotis sodalis* (Boyles et al., 2008), as well as for estimating the thermal niche during torpor or euthermia (Landry-Cuerrier et al., 2008). Moreover, previously described patterns of thermoregulation in torpor (Geiser and Baudinette, 1987; Hiebert, 1990; Richter et al., 2015) were carried out through open flow respirometry, a technique that has been crucial for the development of comparative and ecological physiology (e.g., Kleiber, 1932; McNab, 1986; Glazier, 2005; Lighton, 2008), but which at the same time impose important limitations for analyzing free-ranging animals. The results presented here predicting metabolic changes during torpor (see also Humphries et al., 2002; Rezende and Bacigalupe, 2015; Menzies et al., 2020; Wolf et al., 2020) show that bioenergetic models sometimes replace costly laboratory methods and that the use of miniature data loggers in some way allows “animals to perform the experiment.”

### Conclusion

The Monito del Monte represents the sister lineage of the monophyletic Australidelphia (= Australasian marsupials, Mitchell et al., 2014), exhibiting many of the characteristics of a protoendotherm, and can be considered as a “basoendotherm” (i.e., the endothermic characteristics of Cretaceous and Early Cenozoic mammals, *sensu* Lovegrove, 2012). According to Lovegrove (2012), the resting, normothermic *T*_B_ range of basoendotherms is below 35°C, with a variation range of 5°C, which is the case for monitos, which can have 10°C of *T*_B_ variation in euthermia (Cortes et al., 2009; Nespolo et al., 2010). Given that homeothermy in mammals could have evolved in hot climates through an ancestral heterothermal state (Nowack et al., 2020), early mammals may have relied primarily on passive body temperature regulation with the ability to upregulate short- or long-term metabolism when needed. This is consistent with the thermal niche expansion model, which poses that the evolution of endothermy in mammals occurred *via* the shift from nocturnality to diurnally (see Crompton et al., 1978; reviewed in Hayes and Garland, 1995). Then, monitos are excellent models to study the evolution of endothermy, as they represent the living legacy of ancestral basoendotherm-heterotherms.

## Data Availability Statement

The raw data supporting the conclusions of this article will be made available by the authors, without undue reservation.

## Ethics Statement

The animal study was reviewed and approved by the Chilean Agriculture and Livestock Bureau (SAG; permits No 4371/2019 and 3393/2019), and by the Bioethics Committee of the Austral University of Chile (resolution 313/2018 annex 2019).

## Author Contributions

RN conceived the study, designed the methodology, and wrote the first draft of the manuscript. CM and AE contributed to the field and laboratory work, and manuscript editions. ER, FB, and FF contributed with the statistical analyses and manuscript editions. All authors contributed to the article and approved the submitted version.

### Conflict of Interest

The authors declare that the research was conducted in the absence of any commercial or financial relationships that could be construed as a potential conflict of interest.
